# Severe acute pancreatitis with inflammation extending to the scrotum

**DOI:** 10.1002/ccr3.2529

**Published:** 2019-11-06

**Authors:** Koh Fukushi, Keiichi Tominaga, Kazuhiro Takenaka, Fumiaki Takahashi, Kouhei Tsuchida, Toshimitsu Murohisa, Makoto Iijima, Atsushi Irisawa

**Affiliations:** ^1^ Department of Gastroenterology Dokkyo Medical University Tochigi Japan

**Keywords:** case report, scrotum, severe acute pancreatitis

## Abstract

Extension of inflammation into the scrotum is rare in acute pancreatitis. If inflammation spreads in the scrotum, it may become severe. Clinicians should be aware of this condition as a possible complication. Proactive imaging testing is recommended when complaining of cyst swelling or testicular pain.

## Graphical abstract

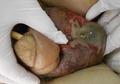

A 74‐year‐old man visited a hospital with left abdominal pain. Blood examination showed an elevated serum pancreatic amylase level 801 U/L and lipase level 580 U/L. CT showed pancreatic enlargement with inflammation extending to the surrounding tissues, and the patient was admitted to the intensive care unit, fortunately, without tracheal intubation or dialysis. In the Japanese severity score,^1^ the prognostic score and CT severity grade indicated “severe” acute pancreatitis. On hospital day 4, scrotal enlargement, black degeneration, and ulcer were observed (Figure 1). CT showed inflammation extending from the retroperitoneum to the surrounding tissues of the left scrotum (Figure 2A,B). Scrotal incision revealed serous drainage and yellow necrotic tissues. We performed debridement. Furthermore, CT revealed retroperitoneal abscess, for which percutaneous retroperitoneal drainage was performed, and on hospital day 13, it was converted into a general ward. However, we could not control retroperitoneal abscess and sepsis, eventually had heart failure and pneumonia. On hospital day 65, the patient died due to sepsis.

**Figure 1:**
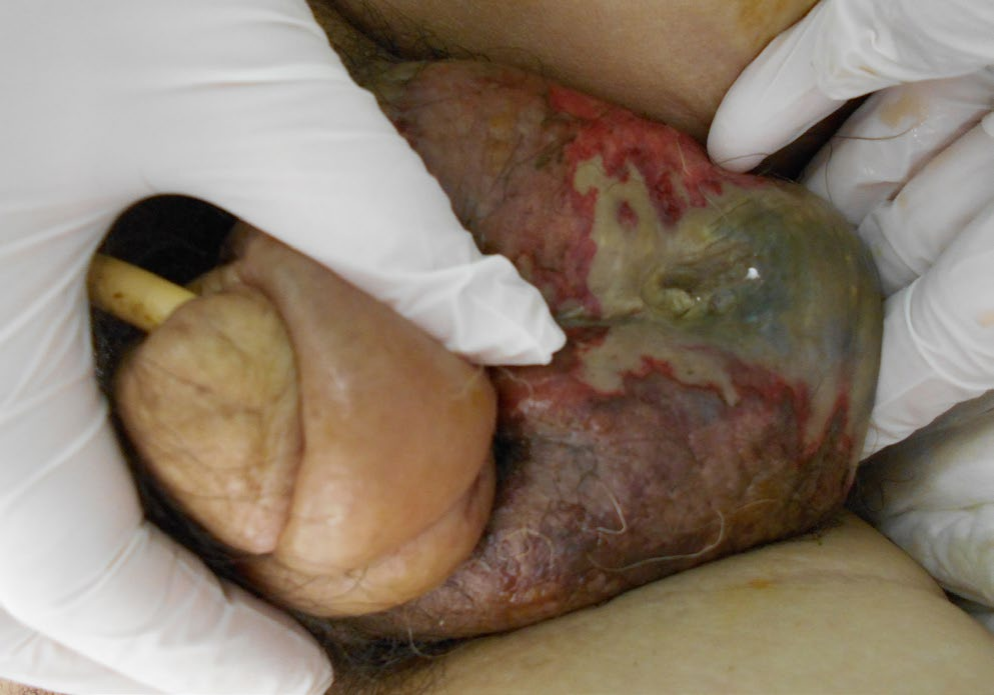
Scrotal swelling, blackened skin, and ulcer are observed

**Figure 2:**
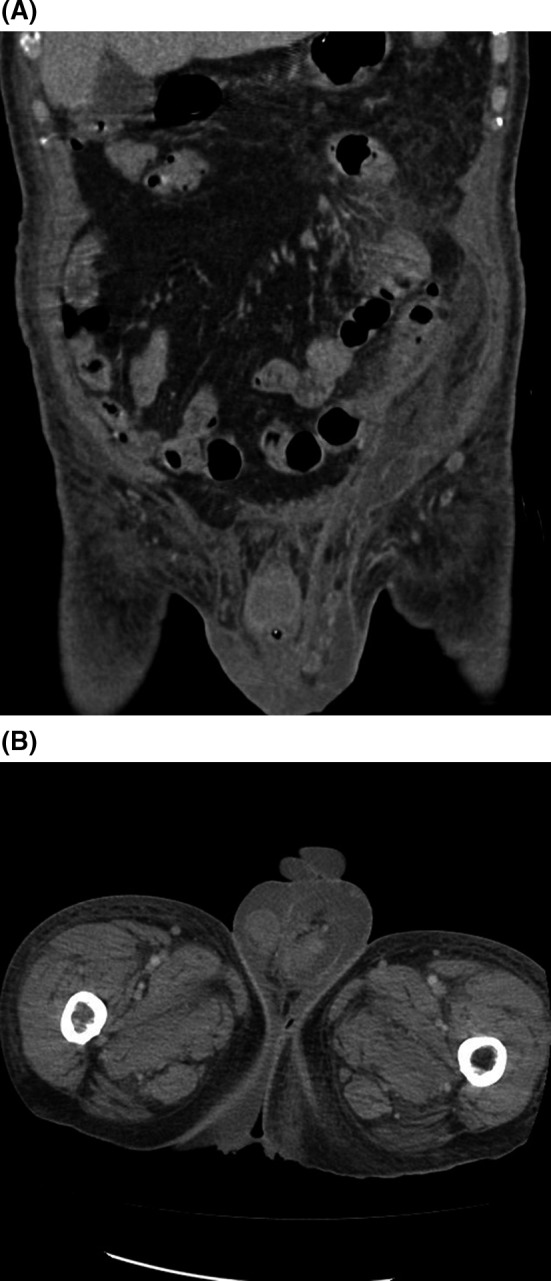
Coronal CT (A) and axial CT (B) show fluid collection in the retroperitoneum, flowing into the left scrotum via the left inguinal canal

The first case of acute pancreatitis with inflammation extending to the scrotum was reported in 1979.^2^ The presence of latent hernia was considered causative factor. Clinicians should be aware of this condition as a possible complication.

## CONFLICT OF INTEREST

All authors declare no conflicts of interest related to this article.

## AUTHOR CONTRIBUTIONS

KF: wrote the manuscript. K Tominaga: reviewed and edited the manuscript. K Takenaka and FT: assisted to the evaluation of the patient's every day condition. K Tsuchida, TM, and MI: supervised the patient's care and management. AI: contributed to critical analysis of the paper.

## INFORMED CONSENT

Ethical approval was not necessary because this study was only a case report.
